# Computer-based multiple component cognitive training in children with ADHD: a pilot study

**DOI:** 10.1186/s13034-022-00553-z

**Published:** 2023-01-16

**Authors:** Yingying Wu, Lingzi Xu, Zhaomin Wu, Xiaolan Cao, Gui Xue, Yufeng Wang, Binrang Yang

**Affiliations:** 1 Shenzhen Children’s Hospital, No.7019, Yitian Rd, Shenzhen, 518000 Futian District China; 2 State Key Laboratory of Cognitive Neuroscience and Learning, Beijing Normal University, Beijing, 100875 China; 3 Peking University Sixth Hospital/Institute of Mental Health, National Clinical Research Center for Mental Disorders (Peking University Sixth Hospital), Beijing, China; 4 Peking University, Beijing, China

**Keywords:** Attention-deficit/hyperactivity disorder (ADHD), Cognitive training, Clinical trial, Subgroup analysis

## Abstract

**Background:**

ADHD is associated with deficits in executive functions. Cognitive training is a promising nonpharmacological intervention for ADHD, however, there is insufficient evidence to guide the selection of training for individuals with ADHD. This pilot study aims to investigate the efficacy of executive function training targeting key executive dysfunctions in ADHD, compared with general executive function training which targets other executive functions.

**Methods:**

A total of 127 subjects (6–12 years) diagnosed with ADHD were allocated to receive one of two different cognitive trainings. ADHD symptoms and cognitive functions were evaluated using parent-rated scales and CANTAB cognitive assessments. All participants were required to complete 48 training sessions within a two-month period.

**Results:**

94 out of 127 children completed the required training and assessments. Both ADHD executive function training group and general executive function training group showed significant improvement in ADHD symptoms on the ADHD Rating Scale and in executive function on the assessment of CANTAB. There was no significant difference in improvements between the two groups. Subgroup analysis suggested that children who had ADHD-RS total scores less than or equal to 28 at baseline showed greater improvement following ADHD executive function training.

**Conclusions:**

This study indicates that cognitive training can improve ADHD symptoms and executive function, with no difference in efficacy between targeted and generalized cognitive training. In addition, individuals with lower symptom severity may benefit more from training targeting key ADHD executive dysfunctions.

## Introduction

Attention-deficit/hyperactivity disorder (ADHD) is a childhood-onset neurodevelopmental disorder typically characterized by age-inappropriate inattention and/or hyperactivity-impulsivity [3]. It is one of the most common childhood psychiatric disorders, occurring in 5–10% of all children worldwide [20, 29]. Symptoms of ADHD can improve with age for some individuals, while for about 15% of people with ADHD, symptoms persist into adulthood [27]. Compared to unaffected children, children with ADHD tend to have poorer short and long term outcomes, including lower academic achievement [4] and low self-esteem [11]. Further, people with ADHD are at an increased risk for comorbid disorders such as oppositional defiant disorder (26.1%), separation anxiety disorder (16.7%), specific phobia (14.7%), enuresis (14.4%) and obsessive compulsive disorder (10.7%) [25]. These factors contribute to the heavy economic burden on families and society caused by ADHD [41].

Treatment strategies for ADHD include pharmacological and non-pharmacological interventions. Pharmacological treatment for ADHD is highly efficacious and widely prescribed, however unfavorable side effects [38] and concerns over insufficient evidence for long-term benefits [31] have limited medication adherence in the real-world. Therefore, there is a great need to develop safe and efficacious non-pharmacological treatments for children with ADHD.

Cognitive training, which targets executive function deficits associated with ADHD, has been investigated as a potential non-pharmacological treatment of ADHD. Executive function is generally thought to include three core components: inhibition control, working memory and cognitive flexibility; and higher-order cognitive processes such as reasoning, problem solving and planning [8]. Previous studies have shown that dysfunctions in inhibition control, working memory and cognitive flexibility are closely related with ADHD symptoms [18]. Executive dysfunctions in ADHD have far ranging impacts, leading to poor academic performance [5], social function impairment and poor interpersonal relationships [28], which cannot be fully addressed with medication intervention alone [30, 33]. Cognitive training may improve executive dysfunctions in people with ADHD through learning-directed brain plasticity [35]. Specially, it has been reported that cognitive training can modulate neuroplasticity resulting in increased grey matter and cortical volume [36], which could promote cognitive enhancement.

While traditional cognitive training uses a pen-and-paper method and relies on in-person therapy with a trained professional, in recent years studies have increasingly validated computerized cognitive training for people with ADHD [34]. Computerized working memory training has been shown by multiple studies to improve working memory performance [2], but not ADHD symptoms or other executive functions, possibly because children with ADHD suffer from multiple executive function deficits [12] which cannot all be addressed by single domain training. One cognitive training review suggests that multiple component training which focuses on multiple neuropsychological domains may enhance the transfer of cognitive improvements to symptoms and behaviors [7]. However, there is a lack of empirical evidence on the effects of different training paradigm combinations targeting various neuropsychological domains in children with ADHD. In addition, most studies focus on training targeting three key executive function components, but few studies have investigated the effect of training of other executive functions. Therefore, there is currently insufficient evidence to guide the development of multiple component training for ADHD.

Recently, it has been found that transfer effect of working memory training is significant not only in working memory but also in attention and real-life behaviors [22], supporting the case for both near and far transfer of cognitive training. Based on transfer effect and brain network theory [23], we hypothesize that core executive dysfunctions of ADHD could be improved by training targeting multiple cognitive domains. We also hypothesize that non-targeted training can result in improvement through transfer effect, but the effect will be weaker than targeted training. In this paper, we will introduce a pilot study to investigate the effects of two different multiple component trainings in children with ADHD. The first is targeted ADHD executive function training (AET) (Infinite Brain Technology, Beijing, China), which is an intensive cognitive training with adaptive difficulty increments focusing on executive functions impaired in ADHD, including working memory, inhibition control and attention. The second is a general executive function training (GET) that targets other cognitive functions which have not been reported to be specifically correlated with ADHD, such as processing speed, reasoning, and planning.

The primary objective of this study was to compare the effects of multiple component training with different executive function combinations on ADHD symptoms and cognitive dysfunctions. In addition, the efficacy and safety of at-home digital cognitive training intervention for eight weeks with ADHD children will be discussed.

## Methods

This study was a clinical trial conducted from April 2021 to January 2022 at Shenzhen Children’s Hospital. This study was approved and reviewed by the Ethics Committee in Shenzhen Children’s Hospital.

### Participants

**Fig. 1:**
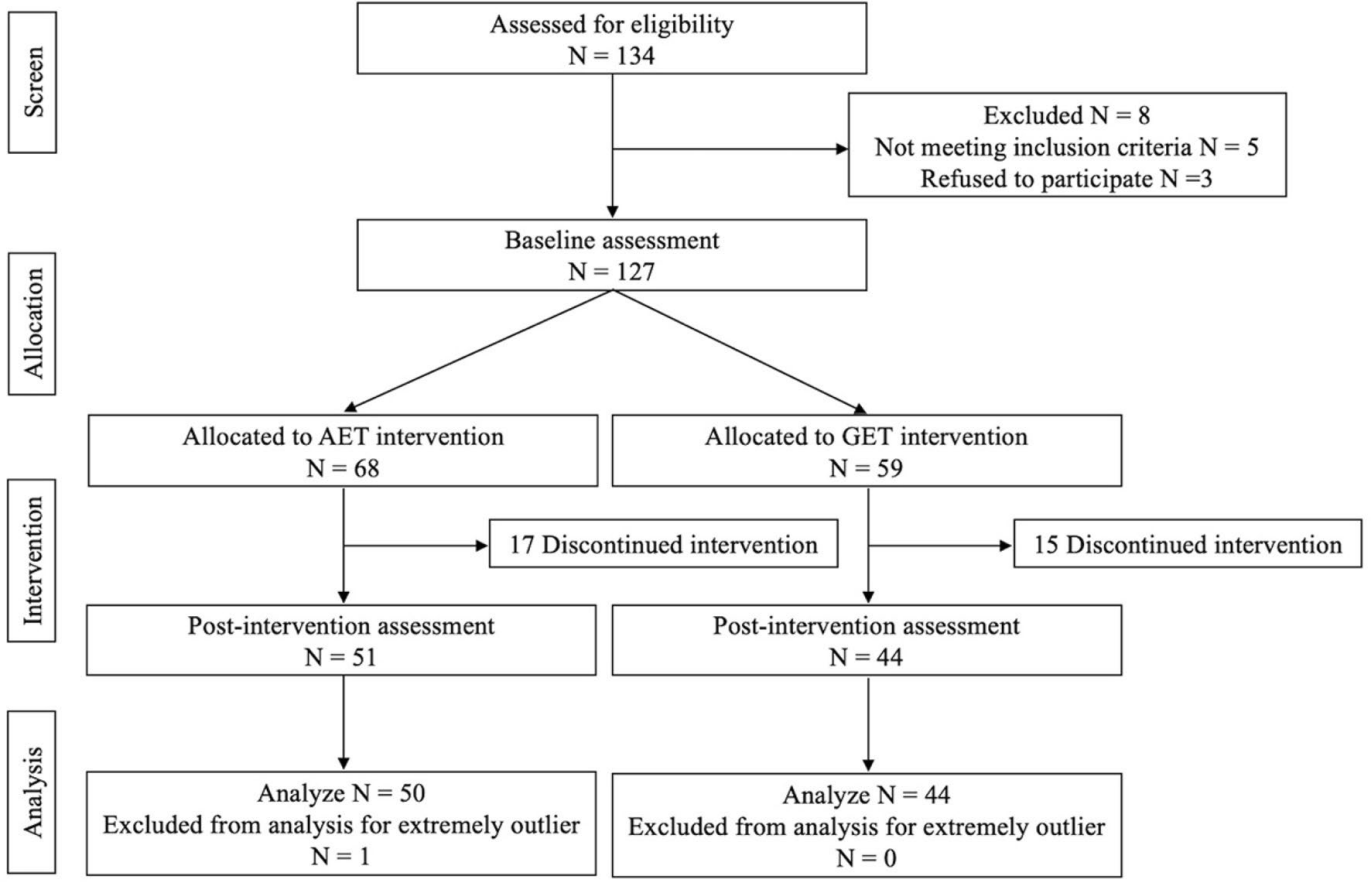
Flow diagram of the clinical trial

Participants were recruited from outpatient clinics at Shenzhen Children’s Hospital. From previous literature review [9, 16] and preliminary user test data, we expected that the drop-out rate of AET would be higher than that of GET. Therefore, eligible participants were allocated in a 1.2:1 ratio to receive ADHD executive function training or a general executive function training (Fig. 1).

ADHD children aged 6 to 12 years old were diagnosed by child psychiatrists according to the Diagnostic and Statistical Manual of Mental Disorders (DSM-IV) and the diagnoses were confirmed using Kiddie Schedule for Affective Disorders and Schizophrenia for School-Aged Children Present-Lifetime version (K-SADS-PL parent version) [24]. Other eligibility criteria include IQ of 80 or above on the Chinese version of Wechsler Intelligence Scale (fourth edition) for Children (WISC-IV) [40] and ability to understand the tasks in the training. Exclusion criteria were as follows: (1) serious medical conditions or neuropsychiatric diseases such as epilepsy and mental retardation; (2) diagnosis of tic disorders; (3) abnormalities in other medical tests that investigators consider to be clinically significant; (4) initiating or terminating behavioral therapy within the prior three months; (5) use of psychotropic medication in the prior month; and (6) history of gaming addiction. Written informed consent was obtained from all parents or guardians, as well as from participants themselves if they were 8 years old or older before baseline screening. Parents and children were informed that they would be assigned one of two cognitive trainings, and that this study aimed to compare the effect of these trainings on cognitive development in children with ADHD. Participants were unaware of the difference in training between the two groups.

### Procedures

#### Baseline screening

At baseline, participants completed a computer-based cognitive assessment (Cambridge Neuropsychological Test Automated Battery, CANTAB) [21]. Specifically, they completed 6 tasks that assessed cognitive functions often impaired in ADHD, including spatial working memory (SWM), rapid visual information processing (RVP), stop signal task (SST), reaction time (RTI), paired associated learning (PAL) and multitasking test (MTT).

At the same time, parents completed the following questionnaires:

(1) ADHD-Rating Scale-4th edition (ADHD-RS-IV) [26] which evaluates ADHD symptoms in children. Total scores and inattention and hyperactivity subscale scores were used in the analysis.

(2) Behavior Rating Inventory of Executive Function (BRIEF)—Parent Form [14] which evaluates executive function behaviors of children in daily life. Global composite score and sub-scales with metacognition and behavioral regulation were utilized in the current study.

(3) Conner’s Parent Symptom Questionnaire (PSQ) [6] which assesses child behavior problems. The hyperactivity subscale score was used in the analysis.

(4) Strengths and Difficulties Questionnaire (SDQ) [10] which assesses psychosocial problems and strengths in children. The sub-scale with Hyperactivity/Inattention was used in the analysis.

(5) Child Behavior Checklist (CBCL) [1] which assesses behavioral and emotional problems. The sub-scale with Attention Problem was analyzed in the study.

#### Training sessions

After allocation, participants and parents were instructed on software use and training requirements by a remote technical assistant who was unmasked to treatment allocation but did not participate in clinical assessments or communicate with study personnel. Participants were allowed to use either a computer or tablet to undertake the training. Participants in both groups were asked to complete a total of 48 sessions of training within eight weeks at home, or at least 6 sessions of training each week. Each session includes 4 tasks and takes from 25 to 40 min to complete based on individual ability. A reward system which gave out stars based on the child’s performance was utilized to motivate children to engage with the training. Parents would be kindly reminded with a message by the remote technical assistant if their child did not participate in training over 72 h. Post-intervention visits were arranged within one week of completion of 48 training sessions.

#### Intervention conditions

##### ADHD executive function training (AET)

AET, designed and developed by Infinite Brain Technology, is a battery of several digital cognitive trainings designed to improve impaired executive functions related with ADHD, including attention, working memory, and response inhibition. The AET training tasks were adapted from N-back task, visual-spatial memory task, Schulte Grid, Go/ No-go task, and mental calculation. Difficulty is automatically adjusted to match participants’ progressive skills to give them sufficient cognitive stimulation while not being frustratingly difficult.

##### General executive function training (GET)

GET is a multiple component training targeting cognitive functions which are not closely associated with ADHD, such as processing speed, reasoning, and planning. Choice reaction time task, non-symbolic numerical comparison task, path planning task, jigsaw puzzle and continuous performance task are included in the GET training. Required training time and difficulty level adjustment algorithms were the same as the AET group.

#### Statistical analysis

An Intention-To-Treat (ITT) approach was used to compare the treatment effects between the AET group and the GET group. Baseline characteristics and assessment data were summarized using the mean with standard deviation (SD) when appropriate. Normality assumptions were tested using the Shapiro–Wilk test. Baseline assessment scores were compared between the two groups via independent sample t-tests or Mann–Whitney U test if non-parametric. For within-group changes in outcomes, two-tailed paired sample t-tests (or Wilcoxon rank-sum test, if the assumptions for parametric testing are not met) were used. For between group comparison of outcomes, independent sample t-tests (or Mann–Whitney U test, if t-tests are not applicable) were applied to compare changes between groups. Additionally, the standardized mean difference (SMD) was given using Cohen’s *d* effect size.

Posthoc subgroup analysis was conducted using the post–pre difference of ADHD-RS total scores as the outcome variable. Linear regression was first used to select potential variables for subgroup analysis. Then, model-based recursive partitioning (MOB) [39] clustering analysis was applied to those variables to identify the subgroup that could benefit most from AET. A modified bootstrapping method was used to validate the stability of the subgroup condition. The SMD was provided as the ratio of the post–pre mean difference in two groups on the condition given by MOB.

All statistical tests were conducted assuming two-tailed contrasts and the alpha significance level at 0.05. Bonferroni corrections were used to counteract multiple comparison problems, only *p*-values < 0.0017 [0.05/19] were considered significant in the within-group and between-group analyses. All statistical analyses were performed using R statistical software version 4.1.1.

## Results

The participant flow chart is presented in Fig. 1. A total of 134 participants were screened for eligibility. At baseline, 3 participants refused to conduct training and 5 participants did not meet the inclusion criteria. As a result, a total of 127 participants were allocated, resulting in 68 participants in AET group and 59 participants in GET group. Of the 127 participants who were enrolled, 32 (25.19%) failed to complete the training or did not return for post-intervention assessment (Fig. 1). Among them, 17 out of 68 (25%) and 15 out of 59 (25.42%) participants were in the AET group and the GET group, respectively. A total of 95 participants completed all training and were included in the data analysis.

As shown in Table 1, there is no significant difference in demographic and clinical variables between the two treatment groups at baseline. In terms of scales, there are significant improvements in ADHD-RS, BREIF, PSQ and SDQ in both groups (Table 2). As presented in Table 3, there is no significant difference in improvement between the two groups. Similarly, as shown in Table 4, key measures on the CANTAB assessment (between errors in SWM, reaction time in SST, RVPA in RVP, five-choice reaction time in RTI, simple reaction time in RTI, total errors in PAL) improved significantly after training for both groups, but there is no significant difference in improvement between the two groups (Table 5).

**Table 1:** Demographic information and clinical characteristics of participants

	AET group	GET group	*p-*value
	Mean (SD)	Mean (SD)	
N	50	44	
Age, years	8.2 (1.31)	8.5 (1.21)	0.20
Male gender, n (%)	43 (86.0%)	37 (84.1%)	0.80
ADHD-RS total score	31.2 (8.20)	31.9 (8.41)	0.70
ADHD-RS inattention score	17.3 (4.50)	18.2 (3.79)	0.33
ADHD-RS Hyperactivity/impulsivity score	13.9 (5.30)	13.8 (6.09)	0.89
WISC-IV	95.9 (9.06)	93.2 (8.08)	0.13

*SD* standard deviation, *AET * ADHD executive training, *GET *general executive training, *WISC-IV* chinese version of wechsler intelligence scale fourth edition

p values are from a t-test (between-subjects, 2-tailed) comparing the AET group to the GET group

**Table 2:** Descriptive statistics of scales and within-group analysis

Scales	Groups	Preintervention	Postintervention	*p-*value
		mean (SD)	mean (SD)	
ADHD-RS total score	AET	31.2 (8.20)	26.3 (8.37)	< 0.01
	GET	31.9 (8.41)	25.7 (7.34)	< 0.01
ADHD-RS inattention	AET	17.3 (4.50)	14.4 (4.5)	< 0.01
	GET	18.2 (3.79)	14.9 (4.63)	< 0.01
ADHD-RS hyperactivity/impulsivity	AET	13.9 (5.30)	11.8 (5.13)	< 0.01
	GET	13.8 (6.09)	10.8 (4.61)	< 0.01
BRIEF-global executive Composite	AET	2.1 (0.30)	1.9 (0.33)	< 0.01
	GET	2.1 (0.29)	1.9 (0.26)	< 0.01
BRIEF-metacognition	AET	2.3 (0.31)	2.1 (0.35)	< 0.01
	GET	2.3 (0.29)	2.2 (0.30)	< 0.01
BRIEF-behavioral regulation	AET	1.7 (0.38)	1.6 (0.38)	< 0.01
	GET	1.7 (0.34)	1.6 (0.29)	< 0.01
PSQ-hyperactivity index	AET	1.2 (0.54)	1.1 (0.54)	< 0.01
	GET	1.3 (0.47)	1.0 (0.40)	< 0.01
SDQ-hyperactivity/inattention	AET	7.8 (1.77)	7.1 (1.47)	< 0.01
	GET	8.0 (1.61)	7.0 (1.76)	< 0.01
CBCL-attention problem	AET	8.6 (2.22)	8.0 (2.35)	< 0.01
	GET	9.2 (2.28)	8.3 (2.30)	< 0.01

*SD* standard deviation, *AET * ADHD executive training, *GET *general executive training

p values are from a t-test (within-subjects, 2-tailed) comparing the pretest to the posttest

**Table 3:** Descriptive statistics of scales and between-group analysis

Scales	AET	GET	*p-*value	Effect size
	Mean (SD)	Mean (SD)		Cohen’s d (95% CI)
ADHD-RS total score	− 5.0 (8.24)	− 6.2 (7.56)	0.45	0.16 (−0.25,0.57)
ADHD-RS inattention	− 2.9 (4.81)	− 3.2 (4.49)	0.72	0.07 (−0.34,0.49)
ADHD-RS hyperactivity/impulsivity	− 2.1 (4.40)	− 3.0 (4.09)	0.31	0.21 (−0.2,0.62)
BRIEF-global executive composite	− 0.1 (0.20)	− 0.2 (0.21)	0.48	0.15 (−0.27,0.56)
BRIEF-metacognition index	− 0.2 (0.24)	− 0.2 (0.23)	0.53	0.11 (−0.30,0.53)
BRIEF-behavioral regulation index	− 0.1 (0.20)	− 0.2 (0.23)	0.45	0.16 (−0.25,0.57)
PSQ-Hyperactivity index	− 0.2 (0.42)	− 0.3 (0.31)	0.26	0.23 (−0.18,0.64)
SDQ-hyperactivity/inattention	− 0.7 (1.76)	− 1.1 (1.49)	0.42	0.20 (−0.21,0.62)
CBCL- attention problem	− 0.67 (1.63)	− 1.0 (1.86)	0.37	0.19 (−0.23,0.6)

*SD* standard deviation, *AET * ADHD executive training, *GET *general executive training

p values are from a t-test (between-subjects, 2-tailed) comparing the AET group to the GET group

**Table 4:** Descriptive statistics of CANTAB key variables

Measure of CANTAB	Groups	Preintervention	Postintervention	*p-*value
		Mean (SD)	Mean (SD)	
SWM				
Between errors	AET	20.8 (8.09)	14.2 (7.80)	< 0.01
	GET	21.0 (7.28)	15.4 (7.96)	< 0.01
Strategy	AET	8.4 (2.00)	7.7 (2.41)	0.09
	GET	8.5 (2.01)	7.9 (2.37)	0.16
SST				
Reaction time	AET	369.8 (104.67)	291.5 (71.71)	< 0.01
	GET	395.2 (82.15)	313.2 (88.87)	< 0.01
RVP				
A’	AET	0.7 (0.08)	0.8 (0.06)	< 0.01
	GET	0.7 (0.09)	0.8 (0.05)	< 0.01
Response latency	AET	592.6 (231.06)	608.2 (167.52)	0.74
	GET	593.0 (200.89)	548.8 (148.29)	0.42
RTI				
Five-choice reaction time	AET	508.8 (101.25)	462.25 (64.69)	< 0.01
	GET	483.9 (77.71)	459.7 (73.94)	< 0.01
Simple reaction time	AET	467.5 (100.73)	419.7 (61.48)	< 0.01
	GET	442.3 (128.14)	405.9 (57.61)	< 0.01
PAL				
Total errors	AET	16.4 (13.97)	7.0 (7.57)	< 0.01
	GET	16.5 (12.81)	6.6 (6.05)	< 0.01
MTT				
Incongruence cost	AET	36.37 (55.09)	38.46 (38.72)	0.99
	GET	37.5 (62.07)	55.4 (63.99)	0.17
Multitasking cost	AET	97.8 (238.55)	150.6 (94.87)	0.39
	GET	80.3 (235.05)	147.0 (117.38)	0.55

*SD* standard deviation, *AET * ADHD executive training, *GET *general executive training, *SWM *spatial working memory task, *SST* stop signal task, *RVP *rapid visual information processing task, *RTI* reaction time task, *PAL* paired associated learning task, *MTT* multitasking test task

p values are from a t-test (within-subjects, 2-tailed) comparing the pretest to the posttest

**Table 5:** Descriptive statistics of key CANTAB measures and between-group analysis

Measures of CANTAB	AET	GET	*p-*value	Effect size
	Mean (SD)	Mean (SD)		Cohen’s d (95% CI)
SWM				
Between errors	− 7.1 (9.82)	− 5.5 (7.53)	0.43	− 0.18 (−0.63,0.27)
Strategy	− 0.71 (2.48)	− 0.6 (2.60)	0.83	− 0.05 (−0.5,0.4)
SST				
Reaction time	− 73.9 (111.81)	− 80.4 (108.21)	0.82	0.06 (−0.39,0.51)
RVP				
A’	0.08 (0.08)	0.07 (0.08)	0.67	0.10 (−0.35,0.54)
Response latency	13.7 (252.52)	− 26.9 (212.10)	0.44	0.17 (−0.27,0.62)
RTI				
Five-choice reaction time	− 46.8 (72.81)	− 18.71 (68.04)	0.06	− 0.40 (−0.85,0.06)
Simple reaction time	− 52.8 (84.43)	− 38.9 (117.75)	0.20	− 0.14 (−0.59,0.32)
PAL				
Total errors	− 10.5 (14.77)	− 9.63 (11.66)	0.89	− 0.06 (−0.51,0.39)
MTT				
Incongruence cost	0.21 (68.73)	20.0 (89.06)	0.28	− 0.25 (−0.71,0.21)
Multitasking cost	60.7 (239.84)	40.2 (200.39)	0.83	0.09 (−0.36,0.55)

*p* values are from a t-test (between-subjects, 2-tailed) comparing the AET group to the GET group

*SD* standard deviation, *AET * ADHD executive training, *GET *general executive training, *SWM *spatial working memory task, *SST* stop signal task, *RVP *rapid visual information processing task, *RTI* reaction time task, *PAL* paired associated learning task, *MTT* multitasking test task

### Subgroup identification

**Fig. 2:**
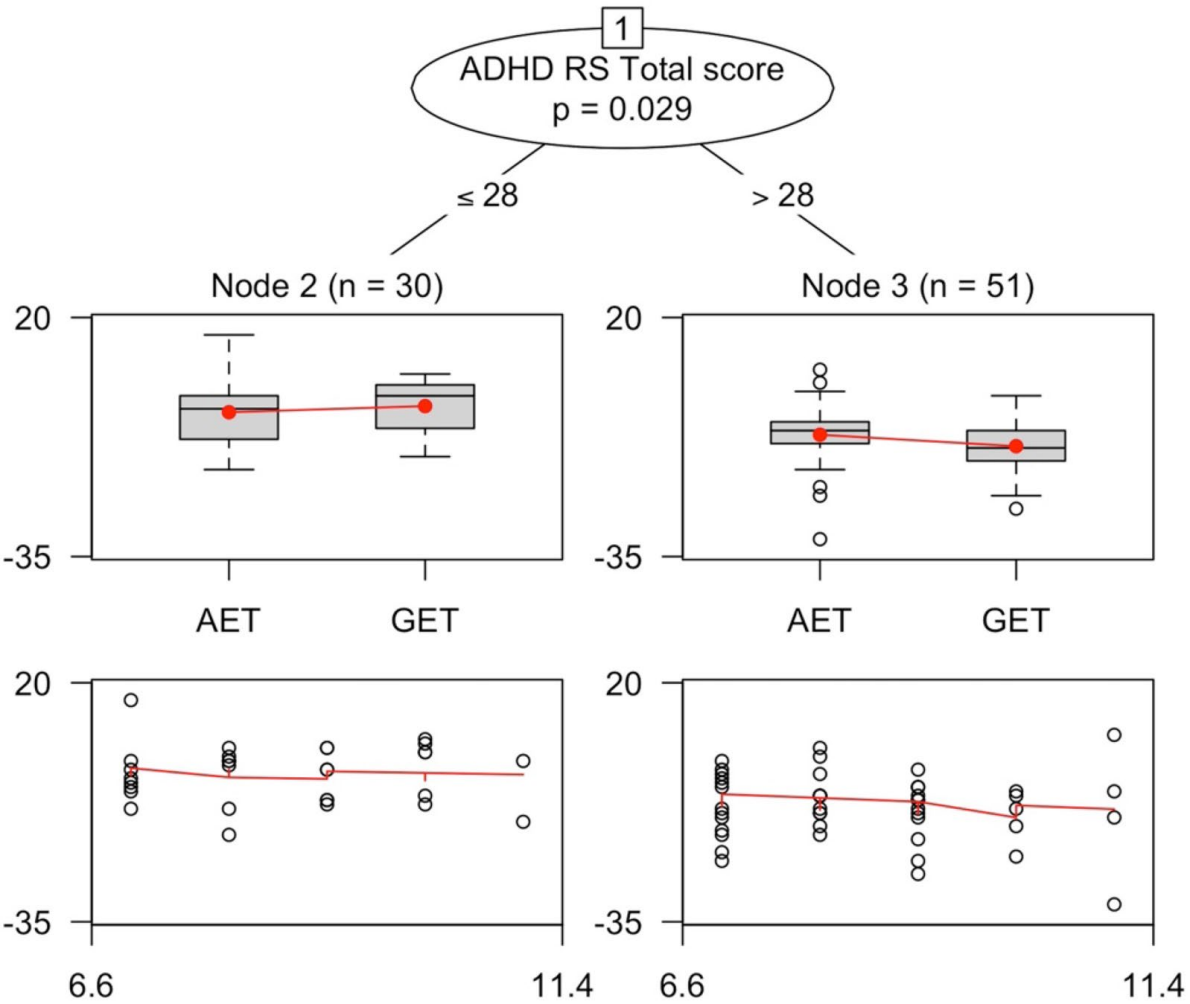
The x-axes in the terminal nodes represent the treatment group, and the y—axes represent the treatment outcome (the ADHD-RS score change after intervention). The scatterplot shows the relationship between age and the treatment outcome. Additional covariates (ADHD-RS total scores, gender, subtype of ADHD, SWMBE, SWMS, RVPA, RVPTH, PALFAMS, MTTICMD, SSTSSRT, RTIFMDMT, RTISMDMT) were used as potential splitting variables, of which one (ADHD-RS total score) was selected

**Fig. 3:**
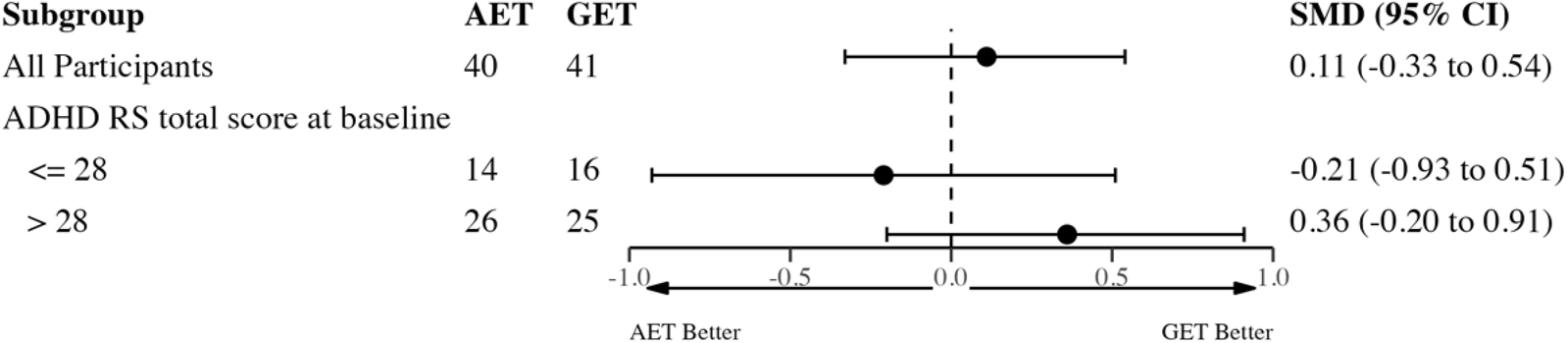
Subgroup results of treatment improvement by ADHD Rating Scale score

In order to further understand whether certain subgroups of patients responded differently to different types of cognitive intervention, we conducted post-hoc subgroup analyses. The result of the linear regression model suggested that ADHD-RS total scores (*β* = − 0.59, *p* < 0.01) at baseline predicted training performance but age (*β* = − 0.24, *p* = 0.68), gender (*β* = 3.17, *p* = 0.13), and ADHD subtype (*β* = − 5.40, *p* = 0.21) did not. Among all variables collected at baseline, those with correlation coefficient less than 0.3 were selected as partition variables to avoid potential multicollinearity. A linear regression tree was recursively fitted on the dataset with age as a prognostic factor and other potential partition covariates (ADHD-RS total scores, gender, subtype of ADHD, SWMBE, SWMS, RVPA, RVPTH, PALFAMS, MTTICMD, SSTSSRT, RTIFMDMT, RTISMDMT). Figure 2 shows that using the model-based recursive partitioning method, ADHD-RS total score of 28 was the optimal threshold to differentiate the effect of the training. As presented in Fig. 3, participants with a baseline ADHD-RS total score less than or equal to 28 benefits more from the AET training than the GET training (SMD = − 0.21, 95% CI [− 0.93,0.51]), while the GET training paradigm may be more beneficial for participants with baseline ADHD-RS total score higher than 28.

### Safety concerns

Parents and participants were informed to report any safety concerns and discomfort to the study staff during training. Intervention safety and acceptance were asked about at the post-intervention visit. Any concerns and discomfort reported by parents and participants during the study were recorded. One participant reported feeling nervous when the training became challenging. Assessed by the investigator, this was not deemed a safety concern related to the study intervention, as there were previous medical conditions causing this issue. Thus, no serious safety concerns related with training has been reported in either group during this study.

## Discussion

This study was designed to determine the efficacy and acceptability of at-home executive function training for ADHD children and the different effects between different multiple component cognitive trainings. We found that both AET and GET training paradigms significantly improved executive function and clinical symptoms in children with ADHD aged 6–12 years old. Notably, we found an average 5.0 points decrease in the ADHD-RS total scores, or a 15.9% decrease from baseline in the AET training group and around 6.2 points reduction (19.4% change from baseline) in the GET group. In the ADHD medication studies, symptom improvement in clinical setting has been usually defined as 30% decrease in symptoms [32]. While the average improvement in each group has not reach the abovementioned threshold, it is comparable with the 6.2 points improvement previously reported in AKL-T01, an FDA approved cognitive training program for children with ADHD [19], but is lower than that of medication treatment which generally see improvement over 10 points) [15]. Fortunately, there were few adverse reactions during the treatment period, which is a major concern in treatment using stimulant medications [15]. Therefore, we believe that cognitive training has a promising benefit to risk profile for treatment of ADHD in children, especially as an alternative option for families who have contraindications for or are unwilling to attempt pharmaceutical treatment.

When comparing two training paradigms, one focusing on ADHD specific executive dysfunctions and one that does not, we found very little difference in treatment effect between the two groups, suggesting that the effects of comprehensive cognitive training for children with ADHD remains robust despite changes in training component. Therefore, excessive focus on working memory or any single cognitive component seems unnecessary. We assume that higher cognitive functions work as a complex web of interacting components (as discussed in [23], rather than as independent components with clearly defined boundaries. Therefore, training that targets processing speed, reasoning and planning might also engage other executive functions such as working memory and attention through overlapping network interaction [37]. Another possible explanation comes from the near-transfer effects (transfer to related executive function improvement) and far-transfer effects (transfer to other executive function and behavioral development) of cognitive training. One meta-analysis suggested that working memory training might produce far-transfer effects on attention, intelligence and reading skills, and that auditory attention training has a far-transfer impact on inhibition and delayed gratification [22]. The transfer effects of multiple component cognitive training might play a role in improving executive function due to dorsolateral prefrontal cortex connectivity [13]. In our study, both multiple component training interventions, the AET training and the GET training, appear to improve cognitive function and behavioral symptoms in ADHD, potentially through transfer effect or functional network interaction. To confirm this hypothesis, more research about different executive function training paradigms should be explored in the future. Furthermore, ecological executive function and psychosocial function should be measured to determine the transfer effect of cognitive training.

Though the effect of the AET training and GET training seems to be similar, some interesting outcomes have been found upon further subgroup identification analysis. The result indicates that treatment has different impacts on specific subgroups. Individuals with ADHD-RS total scores below 28 could benefit more from AET, while GET is more beneficial for subjects with ADHD-RS total scores above 28. These unexpected results suggest that people with ADHD may not benefit equally from cognitive training targeting key executive dysfunctions in ADHD. In addition, ADHD is characterized by neurocognitive heterogeneity [18], therefore, individuals with different diseases characteristics and severity may benefit more from certain types of training. Further exploration of the effect of different multiple component cognitive training paradigms is crucial for the development of individualized training interventions.

There are several limitations to the current study. First, we did not include a negative control group, therefore we are unable to ascertain the extent to which placebo effects affected the overall treatment effect. The aim of the study, however, is not to validate the treatment efficacy of cognitive training, but to compare the effect of two different multiple component trainings, therefore negative control group is not necessary for our study question. Second, the length of training was not strictly limited in this study, resulting in some participants exceeding the training period of two months. From the results of our linear regression model, we found that training duration was not predictive of ADHD-RS total scores, therefore this issue has limited effect on the study conclusion. Next, we did not investigate the long-term effect of training. Previous studies have shown that cognitive training has long-lasting benefits for children with ADHD upon follow-up [17]. The present study did not include follow up, which limits our ability to compare the effects of the two types of training over a longer time frame.

## Conclusion

In all, the present study investigates the effects of two different computerized multiple component cognitive training in children with ADHD. The present results suggest that different multiple component training targeting various executive dysfunctions may be effective to improve cognitive function and ADHD symptoms. Subgroup identification found that baseline disease severity as measured by ADHD-RS may predict treatment benefit for individuals. These results suggest that different subgroups may benefit from different multiple component training, which warrants further study. Overall, cognitive training is a promising intervention that improves executive function and symptoms in children with ADHD. In order to provide individualized treatment for children with ADHD, more research is required to better understand individual responses to different cognitive interventions.

## Data Availability

The datasets used and/or analyzed during the current study are available from the corresponding author on reasonable request.
